# Scaffold and SAR studies on c-MET inhibitors using machine learning approaches

**DOI:** 10.1016/j.jpha.2025.101303

**Published:** 2025-04-10

**Authors:** Jing Zhang, Mingming Zhang, Weiran Huang, Changjie Liang, Wei Xu, Jinghua Zhang, Jun Tu, Innocent Okohi Agida, Jinke Cheng, Dong-Qing Wei, Buyong Ma, Yanjing Wang, Hongsheng Tan

**Affiliations:** a Clinical Research Institute & School of Public Health, Shanghai Jiao Tong University School of Medicine, Shanghai, 200025, China; b Department of Biochemistry and Molecular Cell Biology, Shanghai Key Laboratory for Tumor Microenvironment and Inflammation, Shanghai Jiao Tong University School of Medicine, Shanghai, 200025, China; c Academy of Integrative Medicine, Shanghai University of Traditional Chinese Medicine, Shanghai, 201203, China; d Engineering Research Center of Cell & Therapeutic Antibody, School of Pharmacy, Shanghai Jiao Tong University, Shanghai, 200240, China; e Core Facility of Basic Medical Sciences, Shanghai Jiao Tong University School of Medicine, Shanghai, 200025, China; f School of Life Sciences and Biotechnology, Shanghai Jiao Tong University, Shanghai, 200030, China

**Keywords:** c-MET inhibitors, Machine learning, Structure-activity relationship, Hierarchical clustering, Scaffold based chemical space, Active cliff

## Abstract

Numerous c-mesenchymal-epithelial transition (c-MET) inhibitors have been reported as potential anticancer agents. However, most fail to enter clinical trials owing to poor efficacy or drug resistance. To date, the scaffold-based chemical space of small-molecule c-MET inhibitors has not been analyzed. In this study, we constructed the largest c-MET dataset, which included 2,278 molecules with different structures, by inhibiting the half maximal inhibitory concentration (IC_50_) of kinase activity. No significant differences in drug-like properties were observed between active molecules (1,228) and inactive molecules (1,050), including chemical space coverage, physicochemical properties, and absorption, distribution, metabolism, excretion, and toxicity (ADMET) profiles. The higher chemical diversity of the active molecules was downscaled using *t*-distributed stochastic neighbor embedding (*t*-SNE) high-dimensional data. Further clustering and chemical space networks (CSNs) analyses revealed commonly used scaffolds for c-MET inhibitors, such as M5, M7, and M8. Activity cliffs and structural alerts were used to reveal “dead ends” and “safe bets” for c-MET, as well as dominant structural fragments consisting of pyridazinones, triazoles, and pyrazines. Finally, the decision tree model precisely indicated the key structural features required to constitute active c-MET inhibitor molecules, including at least three aromatic heterocycles, five aromatic nitrogen atoms, and eight nitrogen–oxygen atoms. Overall, our analyses revealed potential structure-activity relationship (SAR) patterns for c-MET inhibitors, which can inform the screening of new compounds and guide future optimization efforts.

## Highlights

• Largest c-MET dataset uncovers structure-activity patterns.
• Commonly used scaffolds (M5, M7, and M8) identified for c-MET inhibitors.
• Active cliffs reveal pyridazinones, triazoles, and pyrazines as key fragments.
• Key features of active c-MET inhibitors: 3 aromatic heterocycles, 5 aromatic nitrogen atoms, and 8 N−O bonds.

## Graphical abstract

**Figure:**
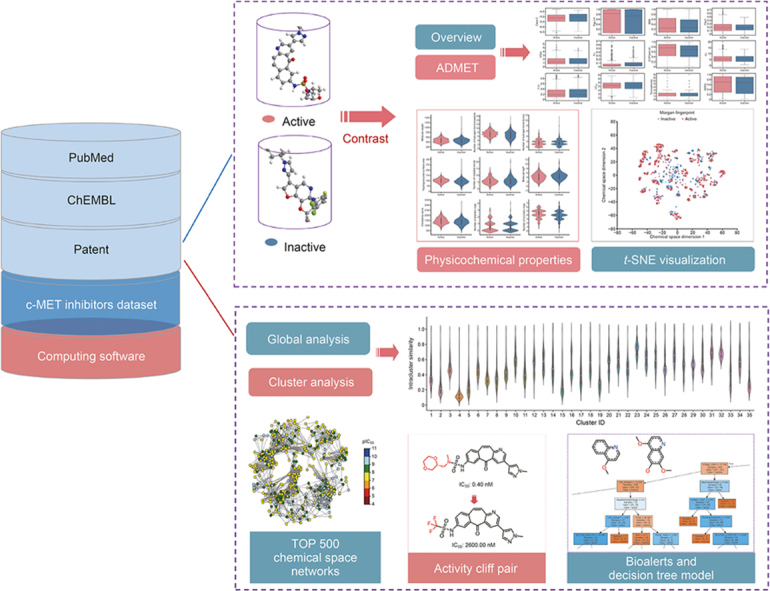

## 1 Introduction

Cancer is a significant global public health issue that poses a severe threat to human well-being. Since the turn of the 21st century, cancer patient outcomes have improved dramatically, with kinase inhibitors playing a pivotal role in tumor-targeted therapies [1,2]. The c-mesenchymal-epithelial transition (c-MET) kinase is a distinct multipotent receptor tyrosine kinase (RTK) with a high natural affinity for hepatocyte growth factor (HGF) [3]. When HGF specifically binds to the c-MET receptor in the extracellular domain, the c-MET protein conformation is altered, forming a dimer. This results in the activation of the protein tyrosine kinase structural domain in the intracellular protein kinase structural sphere [4]. Dysregulation of the HGF/c-MET signaling pathway is closely associated with multiple human malignancies [5,6]. Furthermore, this aberrant activation signaling promotes tumor growth and invasion and is linked to poor prognosis and the acquisition of resistance to treatment [7]. Therefore, c-MET kinase has emerged as a promising target for the development of anticancer drugs.

**Fig. 1:**
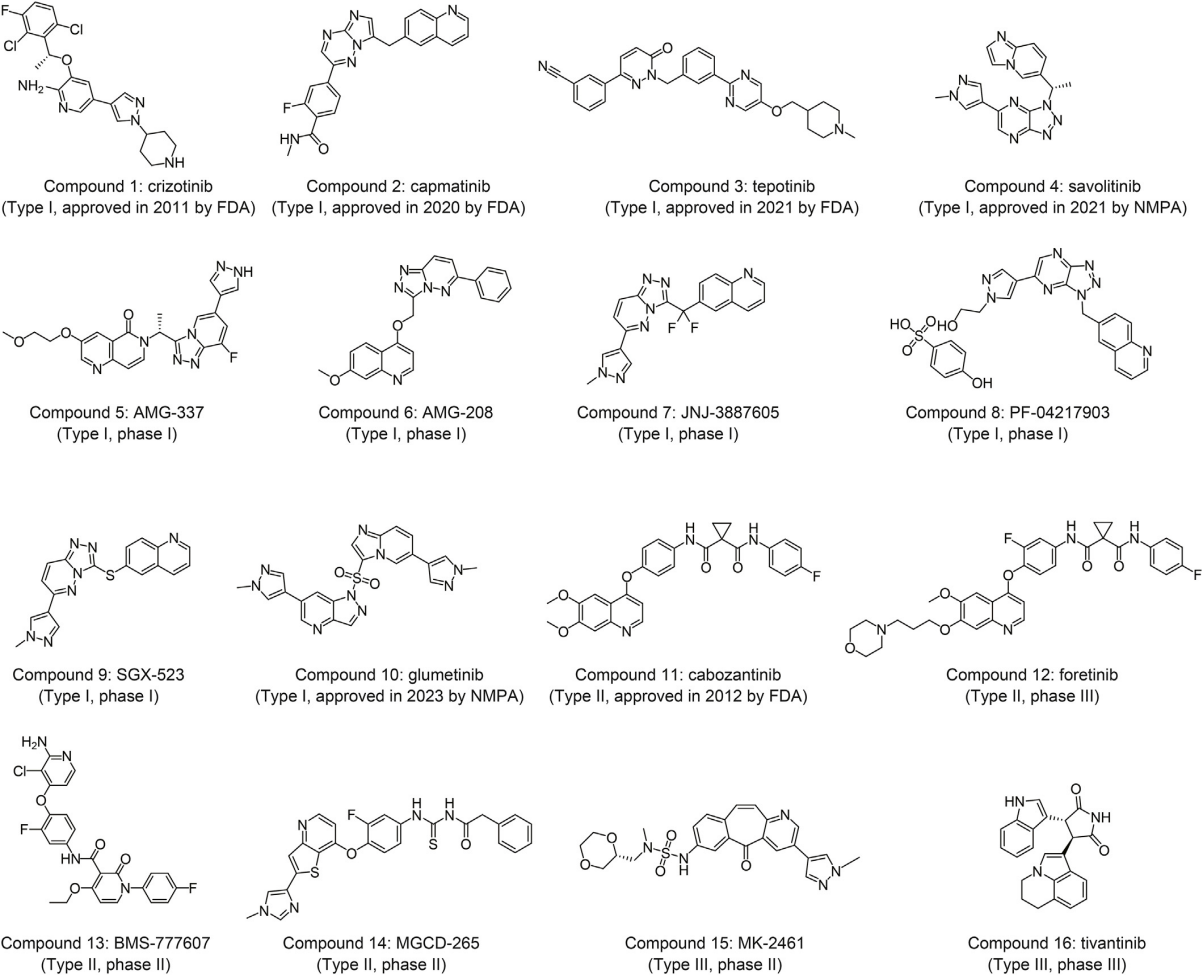
Representative examples of types I, II, and III small-molecule c-mesenchymal-epithelial transition (c-MET) kinase inhibitors launched or in different clinical phase trials. FDA: Food and Drug Administration; NMPA: The National Medical Products Administration, China.

Over the past decade, the development of c-MET inhibitors has undergone remarkable growth. Small-molecule c-Met inhibitors are increasingly regarded as the most promising antitumor agents because of their well-defined mechanisms of action, straightforward synthesis, and ease of modification [8]. Some inhibitors have progressed to the clinical stage or have been marketed. As shown in Fig. 1, c-MET inhibitors can be categorized into three major types (I, II, and III) based on their binding mode to the kinase domain of c-MET. Type I c-MET inhibitors (compounds 1–9) are adenosine triphosphate (ATP) competitive. This type of inhibitor binds to the ATP binding pocket in a U-shaped conformation around Met1211, forming hydrogen bonds with amino acid residues, such as Met1160 and Asp1222 in the c-MET main chain, and forming π–π stacking interactions with Tyr1230 on the A-loop, including crizotinib [9] in Fig. S1. Type II inhibitors (compounds 10–13) are multitarget c-MET and ATP-competitive inhibitors. However, the extended conformational hinges from the solvent-accessible parts to other residues extend to the deep hydrophobic Ile1145 sub-bag near the c-helix region. Foretinib (compound 11), which served as an example, is presented in Fig. S1. Type III inhibitors, including MK-2461 (compound 14) and tivantinib (compound 15), did not conform to the normal c-MET inhibitors. Tivantinib is a non-ATP-competitive inhibitor that binds to the inactive conformation of c-MET, which is beneficial for the stability of self-inhibitory receptors. Despite promising clinical outcomes with c-Met pathway inhibitors, unresolved issues remain, including the lack of specificity of most inhibitors, which leads to inhibitory effects on epidermal growth factor receptor (EGFR) and other kinases, resulting in various clinical side effects. Moreover, the inevitable problem of acquired drug resistance poses significant challenges to c-Met inhibitor development [10,11], highlighting the need for c-Met inhibitors that are more efficient, more selective, and less toxic, featuring novel structures.

An important strategy for proposing new lead candidates involves exploring novel chemical spaces, with emphasis on exploiting structure-activity relationship (SAR) [12,13]. SAR can elucidate the key physicochemical properties and molecular features that underpin a compound’s bioactivity through an exhaustive review of chemical scaffolds and modifications derived from the activity data of established inhibitors [14]. By integrating analytical results with computational modeling techniques, such as three-dimensional SAR models (3D-QSAR) [15] and molecular docking, numerous c-Met inhibitors with desirable properties have been discovered [16]. Machine learning (ML) techniques, such as decision trees, random forests (RFs), and support vector machines (SVMs), have been employed to enhance SAR analysis [17,18]. These techniques are particularly useful for navigating the chemical space of large-scale kinase inhibitor data and facilitating the extraction of meaningful and prioritized structural rules from chemical moieties [19,20]. Over the past few decades, numerous studies on c-MET have revealed diverse scaffold structures in small-molecule inhibitors [21], including triazolopyrazines [22], triazolotriazines [23], quinolones [24,25], pyridine and its fused derivatives [26,27], pyrimidines [28], dioxin pyrazoline [29], and quinoxaline [30]. These core scaffolds are crucial for their inhibitory efficacy and selectivity, providing a starting point for the rational design and optimization of next-generation c-Met inhibitors. However, numerous SAR analyses rely on small datasets for model construction, and relatively few studies have focused on specific SARs within small, low-diversity subgroups. In contrast, large-scale analyses of publicly available accumulated inhibitors and mining of novel information are crucial for understanding the structural patterns of c-MET inhibitors and facilitating rational, evidence-based drug design and discovery.

In this study, we employed cheminformatics and ML to unveil the chemical space, SAR, and structural rule landscape of c-Met inhibitors. A comprehensive dataset comprising 2,278 c-MET inhibitors was compiled to conduct the most thorough analysis to date, with the aim of mining new scaffold information. The maximum common substructure (MCS) of c-MET inhibitors was discovered by hierarchical clustering and chemical spatial visualization, which included the dominant and inactive scaffolds of c-MET inhibitors. Hierarchical scaffold classification was performed using a decision-tree model to derive meaningful activity drivers and rules for the use of c-MET inhibitors. This study highlights the significance of SAR studies in advancing our understanding of c-Met inhibition and guiding the development of more effective lead compounds.

## 2 Experimental

### 2.1 Collection and curation of structure and annotation data for c-MET inhibitors

Small-molecule c-MET inhibitors were primarily derived from ChEMBL (https://www.ebi.ac.uk/chembl/), PubMed, published literature, and patents (https://pss-system.cponline.cnipa.gov.cn/conventionalSearch). The dataset was retrieved from the ChEMBL database by querying the corresponding target database using “UniProt Accessions = P08581” or “ChEMBL ID = CHEMBL3717”. A total of 5,050 molecules in a Simplified Molecular Input Line Entry System (SMILES) format were obtained from ChEMBL 31 (downloaded on December 20, 2022). A total of 74 and 57 molecules were obtained from PubMed and its patents, respectively (from January 1, 2021 to December 31, 2022). We followed the following steps for all small-molecule inhibitors: 1) all SMILES in the dataset were standardized to ensure consistency and facilitate analysis (Chem.MolToSmiles and SaltRemover from RDKit were used to sanitize SMILES and remove salt structures, respectively); 2) manual screening was performed to remove nulls and uncertain extremes; 3) units of c-MET inhibitors were transferred to nM, and uncertain publication years were removed; 4) duplicate data with different labels for data classification were deleted to ensure that the complex appeared only once; and 5) the half maximal inhibitory concentration (IC_50_) of the same compound (e.g., common positive control) was averaged.

### 2.2 Prediction and analysis of absorption, distribution, metabolism, excretion, and toxicity (ADMET) property

ADMETlab 2.0 (https://admetmesh.scbdd.com) was used to predict the ADMET characteristics of active and inactive compounds [31]. The related properties were plotted using Matplotlib to show the distribution of the active and inactive compounds.

### 2.3 Visualization of chemical space using *t*-distributed stochastic neighbor embedding (*t*-SNE)

*t*-SNE is a dimensionality reduction method that preserves the similarity between low-dimensional descriptors and high-dimensional data, making it easy to visualize the high-dimensional features [32,33]. The *t*-SNE implementation in Scikit-learn (https://scikitlearn.org/stable/modules/generated/sklearn.manifold.TSNE.html) was used to compress the original Morgan Fingerprint (1,024 dimensions) into two dimensions. We ran *t*-SNE with default parameters and did not apply any dimensionality reduction before fitting the data.

### 2.4 Visualization of chemical spatial networks using RDKit and NetworkX

Chemical space networks (CSNs) provide a means to visualize and interpret relationships in small-molecule datasets [34]. We created CSNs for the top 500 active molecules ranked by IC_50_ (a smaller value indicates greater activity) using RDKit and NetworkX. First, the data was loaded into an instance of “DataFrame” (from the Pandas package) tailored for CSNs, which include ChEMBL IDs, SMILES, and IC_50_ values. The IC_50_ values were then converted to negative logarithm of IC_50_ (pIC_50_) values (pIC_50_ = −log10(IC_50_)). Subsequently, network edge data were compiled by calculating the pairwise Tanimoto similarity relationships between the compounds. Thereafter, a filter value of ≥0.6 was selected as the threshold for selecting edges to be included in the network. Finally, the node and edge data were added to the network graph. After the network was initialized, it was plotted and optimized by adjusting some network drawing parameters, and the information conveyed by the CSNs visualization was exhibited.

### 2.5 Clustering analysis of structure

To systematically analyze the relationship between chemical structures and bioactivity, we clustered them into a finite number of clusters. First, the SMILES of the compounds were converted to Morgan fingerprints using the GetMorganFingerprintAsBitVect function (radius = 2, bits = 1,024) in RDKit. The agglomerative clustering function implemented in Scikit-learn 0.24.2 was used to perform hierarchical clustering on the dataset. For clustering these molecules, we experimented with the “*n* clusters” parameter, varying it from 20 to 100 while maintaining the other parameters as default (affinity = “Euclidean” and linkage = “ward”). Ultimately, 35 clusters were identified and selected to achieve a satisfactory balance between a reasonable number of clusters with moderate-to-high intracluster similarity. Each cluster was subjected to MCS analysis using the FindMCS function in RDKit. The “ringMatchesRingOnly” parameter was set to "true" to ensure that ring atoms were only matched with each other when comparing different structures. The MCS of a cluster represents the largest substructure shared by all molecules within the cluster, representing a common scaffold. In our analysis, we present the minimum and median intracluster similarities, which correspond to the lowest and middle values between every two compounds within a specific cluster.

### 2.6 Derivation of structural alerts from bioactivity

To understand the relationship between structure and biological activity, the “Bioalerts” package [35] was used to identify substructures that relate to the inhibition activity of c-MET. This method involves counting the occurrence of molecular substructures in active and inactive molecules by employing a specific search radius and analyzing the data using a hypergeometric distribution. If a substructure was significantly more abundant in active molecules than in inactive molecules or vice versa, it was considered an alert structure. In this study, the ECFP4 fingerprint was used with a search radius of three.

### 2.7 Derivation of activity cliffs

In this study, active and inactive molecules were characterized using Murcko scaffolds to illustrate their chemical diversity. To further investigate the activity variations driven by subtle sub structural differences, we retrieved all activity cliffs (defined as compounds with high similarity but significantly different activities) from our dataset [36,37]. To compile a comprehensive list of active cliffs, we performed a similarity search between each active molecule and all inactive molecules and vice versa. A more stringent threshold of 0.6 was chosen in this study relative to the commonly used Tanimoto coefficient (Tc) of 0.5–0.55 [38]. The molecule pairs with Tc above 0.6 and opposite bioactivity were considered active cliffs.

### 2.8 Identification of the structural rules associated with bioactivity

Decision tree-based anchors were computed to extract meaningful structural rules associated with the activity. The Decision Tree Classifier function in Scikit-learn (https://scikitlearn.org/stable/modules/generated/sklearn.tree.DecisionTreeClassifier.html) was employed along with the anchor (or scoped rules) method [39] using the anchor-exp Python library (https://github.com/marcotcr/anchor/). Initially, a decision tree model was trained with a maximum depth of 10 to prevent overfitting, with all other hyperparameters set to their default values. Notably, the model was created solely for pattern extraction and was neither used nor interpreted as predictive. Subsequently, the trained decision tree was subjected to an anchor-generation procedure using a modified version of the Jupyter Notebook available in the anchor-exp’s GitHub repository (https://github.com/marcotcr/anchor/blob/master/notebooks/Anchor/on/tabular/data.ipynb). The features used to build the model were generated by the molecular descriptor module from the RDKit package, resulting in 208 features for each molecule. A precision threshold of 0.80 was applied to the explainer. Feature interactions were used to establish SAR rules. The anchor-exp Python module was used to calculate the anchors derived from the trained decision tree. The anchors represent the “If … then …” (IF-THEN) rules, which can be interpreted as sufficient conditions for a given activity outcome.

## 3 Results and discussion

### 3.1 Curated c-Met dataset and the temporal trends in c-Met inhibitors development

**Fig. 2:**
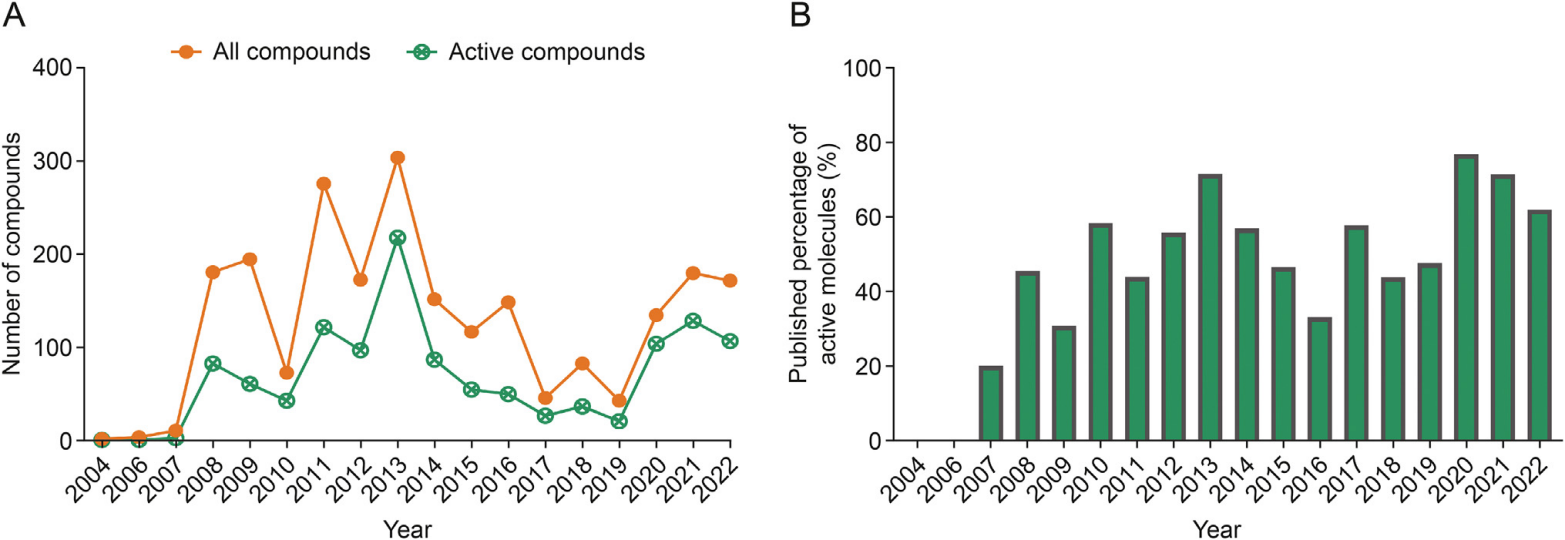
Trends in the number of published small-molecule c-mesenchymal-epithelial transition (c-MET) kinase inhibitors. (A) Number of compounds reported from the year 2004−2022. (B) Histogram of published percentage of active molecules.

To identify structural features associated with inhibitors and facilitate the discovery of novel inhibitors, we first curated a detailed c-Met inhibitor dataset using the collected data, which contained 2,278 molecules in our dataset, including 1,228 actives (IC_50_ ≤ 100 nM) and 1,050 inactive (IC_50_ > 100 nM). Additionally, we examined trends in compound publications over the past few decades. It showed peaks in 2011 and 2013, likely related to the U.S. Food and Drug Administration (FDA) approval of crizotinib in 2011 and cabozantinib in 2012, which fueled research interest in developing c-MET inhibitors (Fig. 2A). Although the number of inhibitors decreased in 2012, the proportion of active compounds increased, indicating that published inhibitors provide a reference for designing c-MET inhibitors. Interestingly, the proportion of active molecules increased after 2009 (Fig. 2B). Nevertheless, the lipophilicity of the compounds, topological polar surface area, and number of hydrogen bond donors and acceptors remained stable (Fig. S2). Bibliometric techniques were used to analyze the literature included in the Web of Science database. Among the journals reporting these compounds, 45.44% are Q1 journals (Table S1), such as *Journal of Medicinal Chemistry* (25.99%) and *European Journal of Medicinal Chemistry* (19.32%), which require the reported compounds to be novel with good *in vitro* and *in vivo* activities, demonstrating innovative aspects.

### 3.2 Physicochemical properties and ADMET analysis based on actives and inactive compounds

**Fig. 3:**
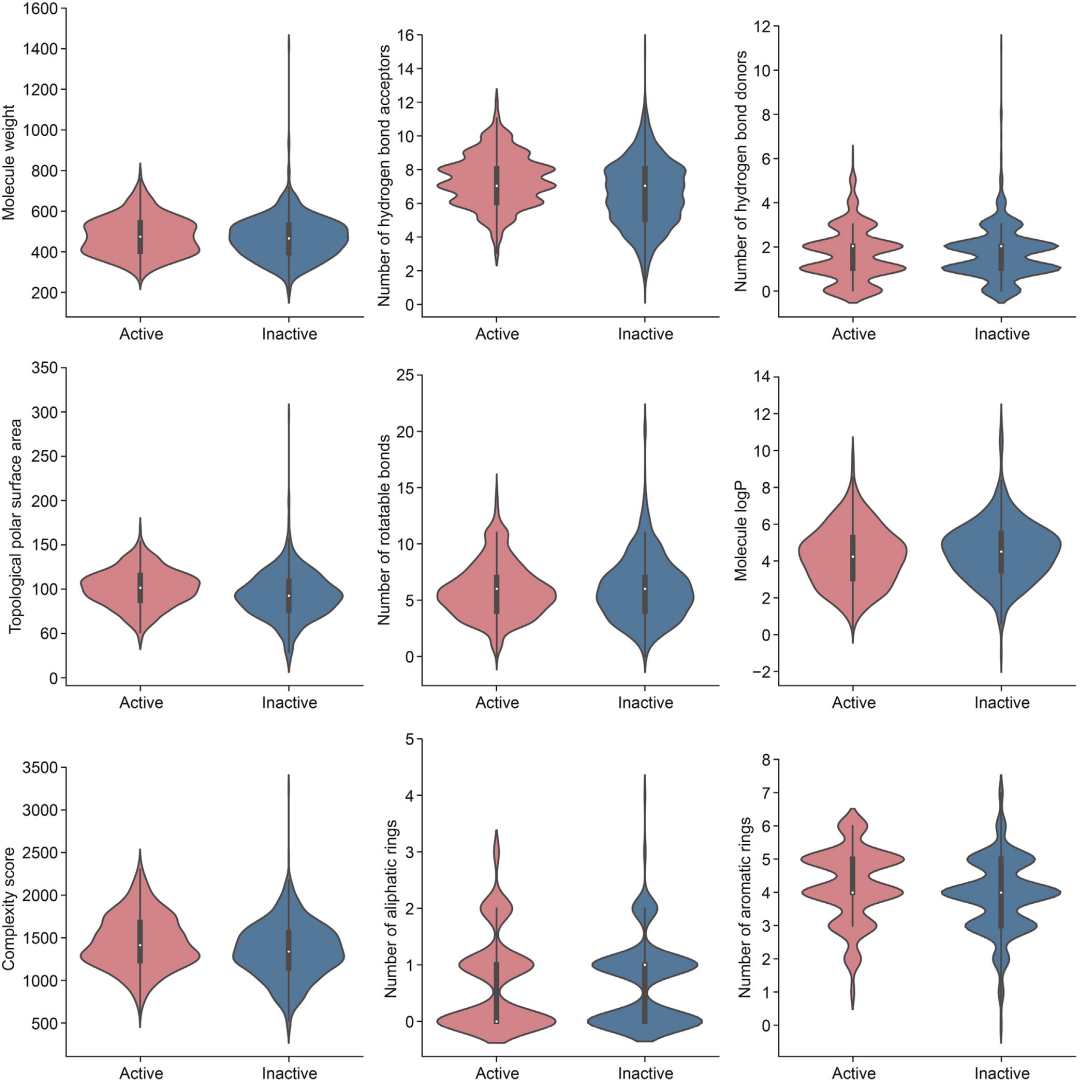
Comparison of the key physicochemical properties of active (red) and inactive (dark blue) compounds. For the different properties, there are almost no major differences between the two classes. logP: logarithm of the partition coefficient.

**Fig. 4:**
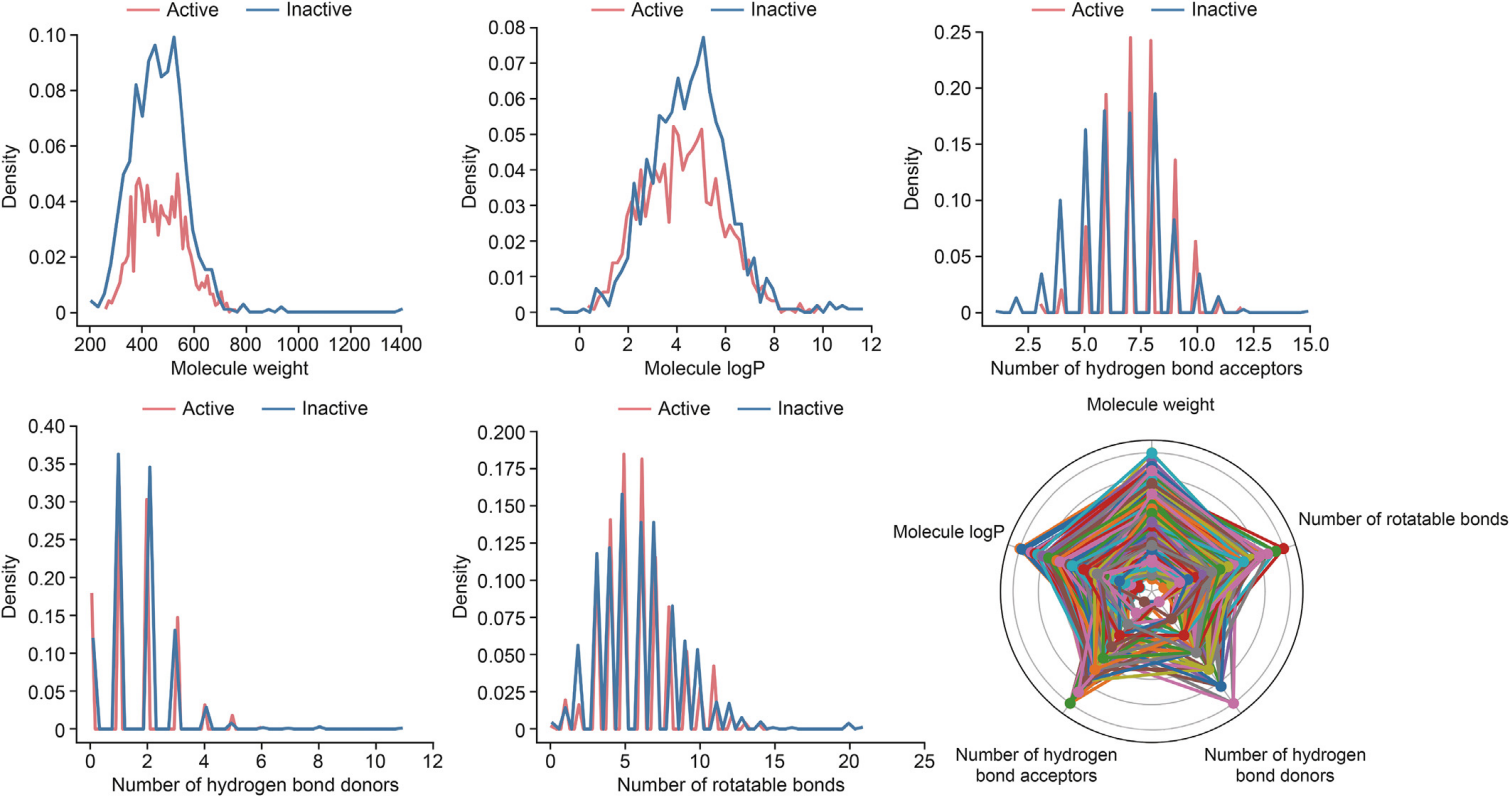
Distribution of c-mesenchymal-epithelial transition (c-MET) active inhibitors (red) and inactive inhibitors (blue) with respect to the four rule-of-five descriptors: molecular weight, logarithm of the partition coefficient (logP), and the numbers of hydrogen bond donors and acceptors.

Physicochemical properties and ADMET are key considerations for medicinal chemists in the design and optimization of lead compounds. The physicochemical properties of the active and inactive compounds were evaluated and analyzed. As shown in Fig. 3, the distributions of active and inactive compounds across the nine evaluated physicochemical properties exhibited no significant differences between the two classes, and the overall distribution of active compounds appeared to be more concentrated than that of the inactive set. Moreover, neither the molecular weight nor logarithm of the partition coefficient (logP) appeared to directly influence activity, as increased lipophilicity enhances activity through nonspecific binding. Its application to both the active and inactive sets revealed that most compounds from both categories fell within the permissible range of the rule (Fig. 4).

Subsequently, the pharmacokinetic parameters of the active and inactive molecules were predicted using ADMET 2.0 [31]. The results (Fig. S3) demonstrated no significant differences between the two sets, although the active molecules exhibited slightly more favorable profiles for certain parameters, such as blood-brain barrier (BBB) permeability and cytochrome P450 3A4 (CYP3A4) inhibition propensity, than their inactive counterparts.

As revealed in this study, for molecules derived from kinase selectivity screening sets, even those exhibiting distinct kinase inhibitory potencies (classified as active or inactive), the physicochemical property space and ADMET profiles were not significantly different between the active and inactive molecules. This suggests that c-MET inhibition is not determined by a specific characteristic, such as logP, but rather by subtle changes or the presence of certain scaffolds and/or substructures. Consequently, subsequent research will concentrate on the cluster analysis of compound scaffolds and active fragments to uncover the molecular basis of c-MET kinase inhibition, offering deeper insights into the design and optimization of c-MET kinase inhibitors.

### 3.3 Chemical space visualization and chemical space analysis

**Fig. 5:**
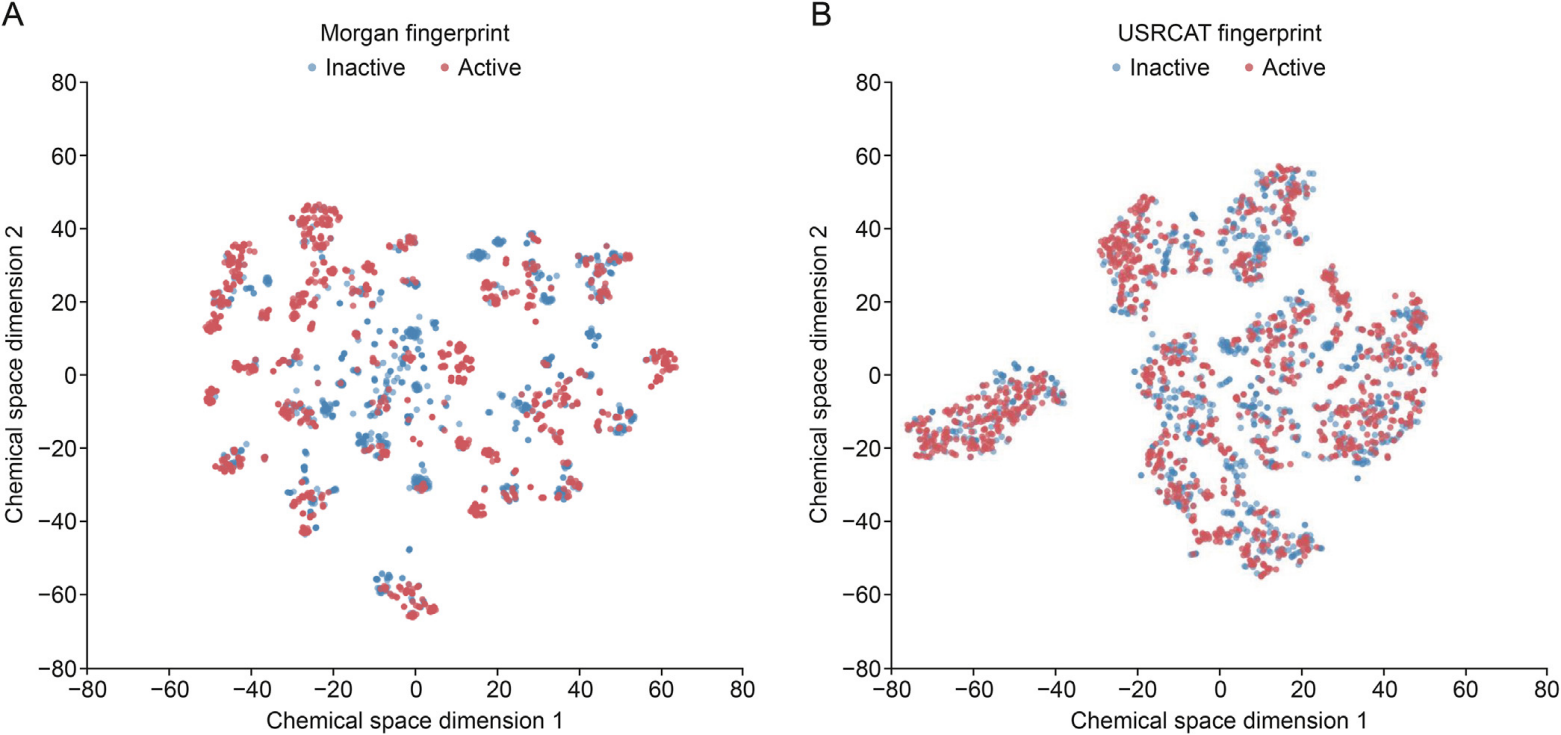
*t*-Distributed stochastic neighbor embedding (*t*-SNE) visualization of the distribution of the (A) Morgan fingerprint and (B) ultrafast shape recognition of CREDO Atom Types (USRCAT) fingerprint in two-dimensional (2D) space.

To elucidate the chemical diversity and spatial distribution of active and inactive molecules, *t*-SNE mapping analysis was conducted using both the 2D Morgan circular fingerprint and 3D Ultrafast Shape Recognition of CREDO Atom Types (USRCAT) fingerprints. As shown in Fig. 5, both the active and inactive compounds exhibited numerous clusters in the 2D Morgan fingerprints (Fig. 5A) and 3D shape fingerprints (Fig. 5B). Particularly in 5A, although the active and inactive compounds show similar distributions, the actives have subtle differences in chemical scaffolds and spatial structures. This suggests that we can conduct a more in-depth cluster analysis and pattern recognition on the c-MET dataset to discover new scaffolds or new SARs.

### 3.4 Clustering analysis to assess trends of activity and overall diversity

In a large dataset, a group of compounds may share multiple substructures that may contribute to c-MET activity and may arise from combinations of different “classical” scaffolds. During the design process of pharmaceutical chemists, similar structures are compared within the same framework. Hierarchical clustering analysis was performed on the entire dataset to examine the distribution of active molecules across different scaffolds, thereby identifying the more favorable scaffolds. The classical approach of evaluating different parameters using profile coefficients was adopted [40], and the silhouette score reached equilibrium after the number of clusters reached 200 (Fig. S4). Considering the size of each cluster and identifying as many common structural features as possible, we manually adjusted the initial number of spontaneous clusters to achieve a balance between small clusters with high intracluster similarity and large clusters with poor cohesion. Finally, 35 clusters were selected for the analysis. A total of 20 of these clusters showed significant cohesion, with an average intra-cluster similarity ≥ 0.5. Clusters 25 and 18 (the smallest clusters) contained 23 and 25 compounds, respectively. Moreover, this study aimed to analyze the MCS within each cluster to identify common scaffolds, which may include previously unreported scaffolds, warranting further consideration. Table S2 shows a summary of all clusters and their statistics. Fig. S5 shows the intra-cluster similarity distribution for each cluster.

#### 3.4.1 Clusters predominantly populated by active molecules

**Fig. 6:**
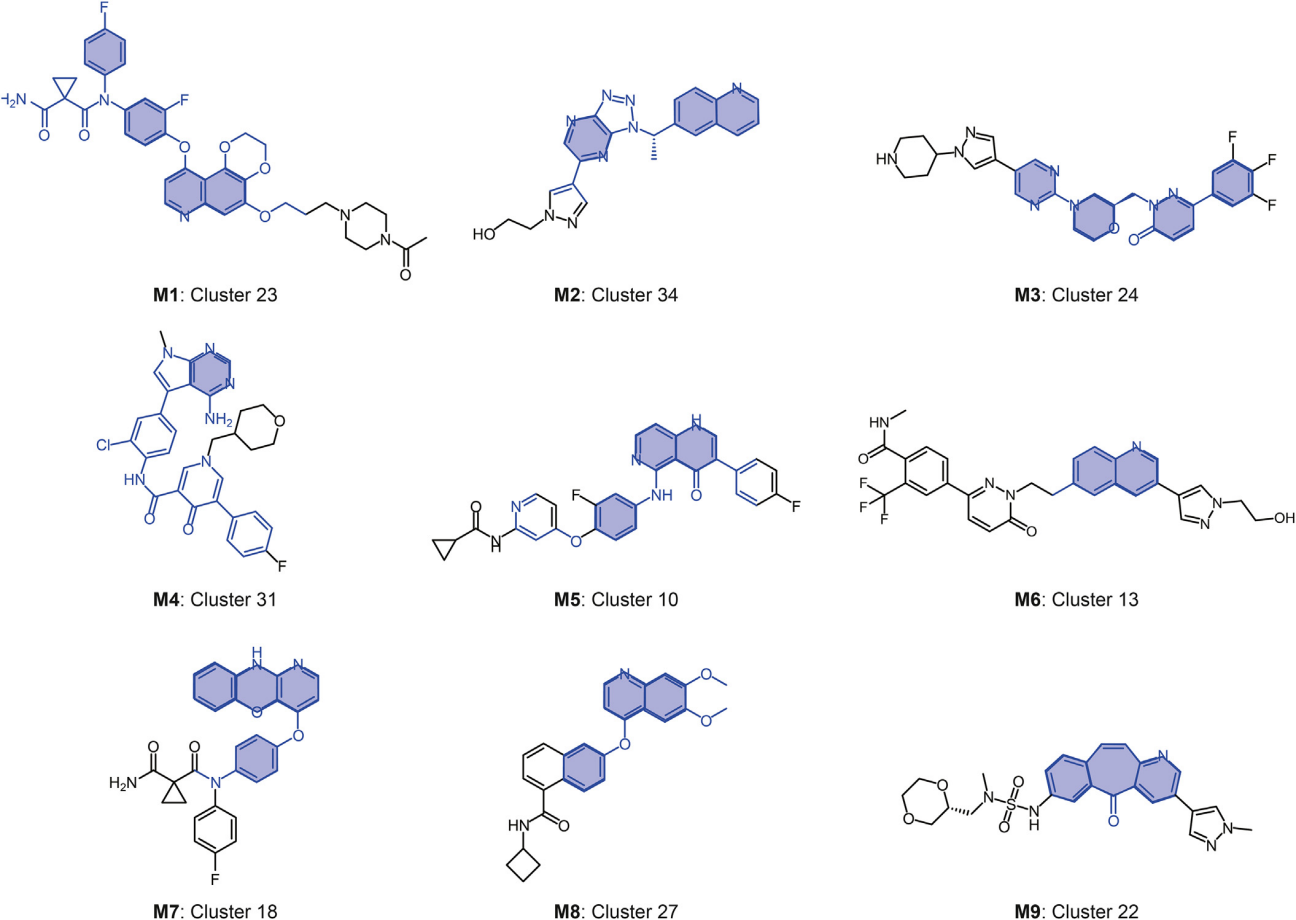
List of scaffolds (blue) with significant variations in activity representing different clusters using exemplary molecules. The highlighted structure indicates the common scaffold obtained through maximum common substructure (MCS) decomposition for each cluster.

Clusters enriched with a higher proportion of active molecules may harbor more meaningful scaffolds. Nine clusters (Cluster IDs: 5, 10, 13, 18, 19, 23, 24, 31, and 34; Table S2) exhibited a high proportion (>80%) of active compounds (383 total). Cluster 23 (Fig. 6, **M1**) comprised 32 active compounds with a high median inhibitory concentration score (ICS) of 0.7 and IC_50_ values below 50 nM. This 100% active cluster suggests that dioxane quinazole is a promising scaffold for c-MET inhibitors. These molecules comprised dioxane quinazolines linked to halogenated phenyls, a skeleton that has also been reported previously [41]. Cluster 34 (Fig. 6, **M2**) consisted of 27 active compounds with a median ICS of 0.57. (*S*)-6-(1-(1*H*-[1,2,3]triazolo[4,5-b]pyrazin-1-yl)ethyl) quinoline was used as the MCS. These active molecules exhibited a maximum IC_50_ of 66.7 nM, demonstrating potent activity in enzymatic and cellular assays, as well as metabolic stability across human, rat, and monkey liver microsomes. Furthermore, these compounds inhibited tumor growth.

Cluster 24 (Fig. 6, **M3**) comprised 30 compounds with a median ICS of 0.63, where 93.3% were active. The characteristic substructures featured phenyl-substituted pyridazin-3-ones substituted with morpholinopyrimidines. Modification of the morpholino pyridazinone scaffold has varying effects on c-MET activity. For instance, replacing the 6-phenyl group with benzonitrile resulted in a 180-fold increase compared to replacement with (benzyloxy)benzene (IC_50_ 4 nM vs. 720 nM). However, replacing benzonitrile with trifluorobenzene, fluorobenzene, or 1-chloro-2-fluorobenzene did not significantly affect activity.

Cluster 31 (Fig. 6, **M4**) consisted of 25 compounds, including 92% active molecules. The MCS of Cluster 31 was *N*-(4-(4-amino-7l2-pyrrolo[2,3-d]pyrimidin-5-yl)-2-chlorophenyl)-4-oxo-5-phenyl-4*H*-1l2-pyridine-3-carboxamide. After SAR analysis, it was found that in the pyrrolo[2,3-d]pyrimidine scaffold, no substituent group was needed between the two nitrogen atoms of the pyrimidine ring, as it did not affect the activity. When a CH_3_ group was connected, the inhibition decreased 66-fold (IC_50_ 2.4 nM vs. 158.9 nM). Moreover, attaching small groups, such as the CH_3_ group, to the nitrogen atom of the pyrrole ring is preferable, whereas long functional groups, such as *N*-dimethylethanamine, significantly reduce the activity.

Cluster 10 (Fig. 6, **M5**) comprised 68 compounds, with a median ICS of 0.59. It includes 91.2% active molecules, with a median IC_50_ of 15.55 nM, indicating good inhibitory effects in the nanomolar range. The dominant scaffold of the c-MET inhibitor in this cluster was hypothesized to be naphthyridinone [42,43]. Similarly, Cluster 13 (Fig. 6, **M6**) included 37 compounds with 89.2% of active molecules. This cluster was characterized by the 6-methylquinoline scaffold, and the molecules within it exhibited potent activity, reaching a low nanomolar range with a minimum IC_50_ of 2.8 nM and a median IC_50_ of 10.6 nM.

Cluster 18 (Fig. 6, **M7**) consists of 25 compounds, comprising 84% of the active molecules. The activity of this cluster reached the low nanomolar range, and the most commonly identified scaffold was 4-((10*H*-benzo[b]pyrido[2,3-e] [1,4]oxazin-4-yl)oxy) aniline. The addition of *N*-methylaniline to 1-acetylcyclopane-1-carboxamide enhances c-MET inhibition. After attaching 1-acetylcycloalkane-1-carboxamide, the group connected to *N*-methylaniline showed 40.35-fold higher than that connected to 5-methylcycloalkane-1-carboxamide (IC_50_ of 3.7 nM vs. 149.3 nM) and 36.81-fold higher than that connected to toluene (IC_50_ 3.7 nM vs. 136.2 nM).

#### 3.4.2 Analysis of clusters with fewer active molecules

Understanding the structural determinants of inactivity in c-MET inhibitors will help avoid unsuccessful compound design. The MCS of Cluster 16 (Fig. 6, **M8**) was 6,7-dimethoxy-4-phenoxyquinoline, which shared a quinoline scaffold with Clusters 13, 23, and 24. Additionally, c-MET inhibitors featuring quinoline scaffolds have been documented [41,44]. In addition, we identified a relatively interesting cluster, Cluster 22 (active = 45.4%), with an active-to-inactive molecular ratio of approximately 1:1 (Fig. 6, **M9**). The span of IC_50_ was also very large (0.2–7,300 nM), among which 30 molecules (30.93%) had IC_50_ ≤ 10 nM. The MCS of Cluster 22 was 5*H*-benzo[4,5]cyclohepta[1,2-b]pyridin-5-one [27]. Because of the large number of molecules in this cluster, we selected the 10 molecules with the strongest activity and 10 molecules with the worst activity for observation. The 7-position connecting group, particularly azanesulphonamide, plays a decisive role in the activity of the scaffold. Various functional groups, including pyrazine, tetrahydrofuran, (*R*)-2-methyl-1,4-dioxane, and 3-methylpyridazine, were connected to the azanesulfonamide. The 2-methylpyridine exhibited potent inhibitory effects on c-MET (IC_50_ ≤ 2 nM) when connected at position 3 via a pyrazole linker. However, the nitrogen atom on the pyrazole moiety had a negligible impact on activity when connected to other small fragments. However, the introduction of a methylsulfonyl substituent at the 7-position resulted in a precipitous drop in activity.

Through cluster analysis, we identified advantageous scaffolds, such as M2, M4, and M7, and analyzed their SAR. However, the analysis of scaffolds, such as M9, indicated that the scaffold was not the sole determinant of c-MET inhibitory activity. For certain specific types of scaffolds, their substructures and chemical fragments composing these scaffolds play a crucial role in influencing the interaction between the compound and the target protein. Therefore, in the design and optimization of c-MET inhibitors, it is essential to carefully explore and adjust the local structural features beyond the overall scaffold selection. This includes, but is not limited to, the type, position, and linkage of functional groups, all aimed at enhancing the drug’s affinity and selectivity towards the target, thereby improving therapeutic efficacy and reducing potential side effects.

### 3.5 CSNs analysis for the top 500 actives

**Fig. 7:**
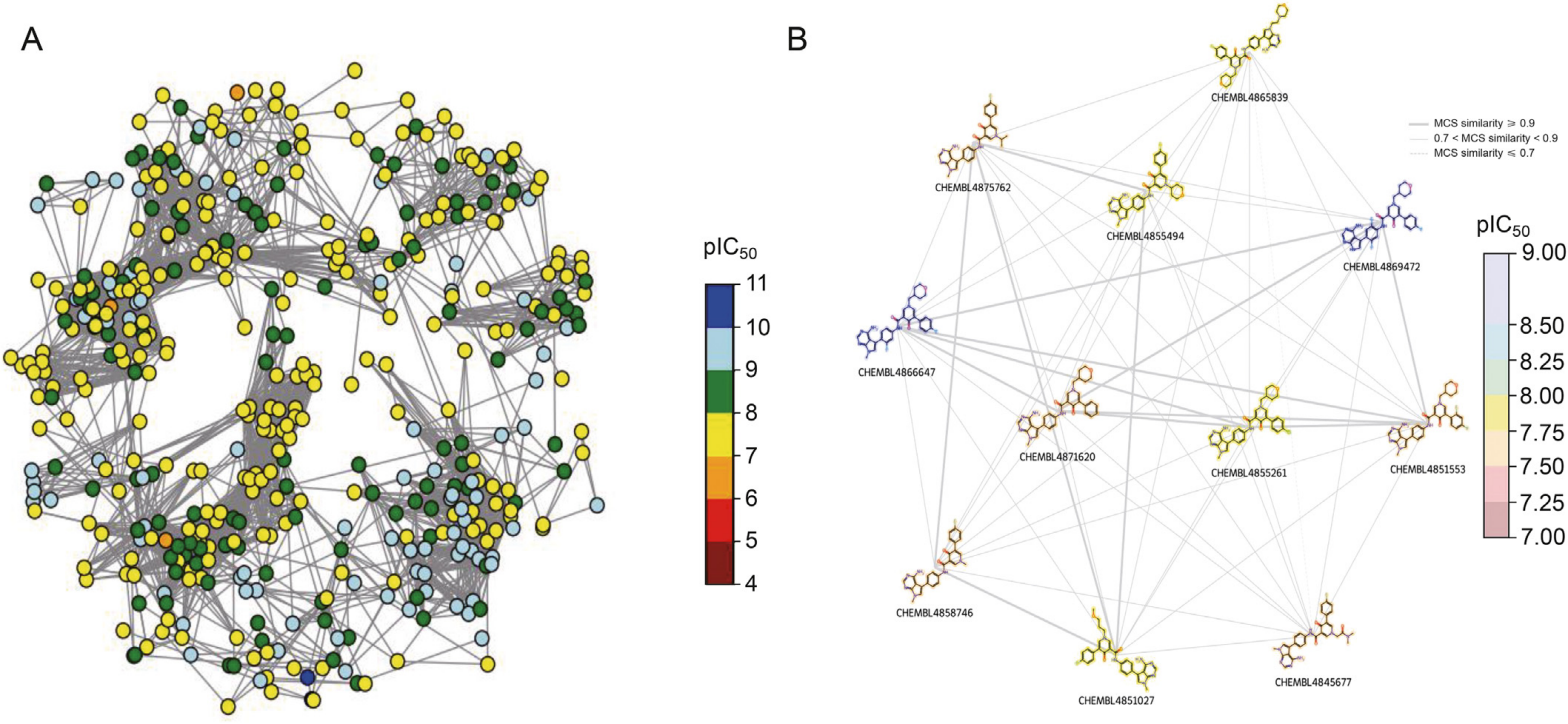
Overview of the entire network and a subgraph with 11 highly similar nodes. (A) Spring layout chemical space networks (CSNs) (Tanimoto similarity variant) with the c-mesenchymal-epithelial transition (c-MET) dataset compounds. The Tanimoto similarity threshold was set to ≥0.6. The node color represents the negative logarithm of the half-maximal inhibitory concentration (pIC_50_) value. (B) Spring layout CSNs component (maximum common substructure (MCS) similarity variant) with the c-MET dataset compounds. The nodes are plotted as two-dimensional (2D) compound images with color highlighting for pIC_50_ values and line style dependent on MCS-based similarity value.

The structural features and scaffold characteristics of active molecules play a pivotal role in drug discovery. As such, we have conducted a comprehensive analysis of the top 500 active c-MET inhibitors to elucidate SAR for the future. This investigation aims to establish a computational and experimental framework for rational drug design, ultimately accelerating the development of innovative therapeutics targeting c-MET pathways. To further analyze and visualize the skeletal features of active molecules, RDKit, NetworkX, and Python were used to demonstrate and visualize molecular connections using similarity as weights [34]. The 500 small-molecule inhibitors with the lowest IC_50_ values were selected. A threshold of 0.6 was used for the MCS similarity analysis, resulting in a network comprising 461 nodes and 4,247 edges, with an edge density of 0.037. In addition, 25 connected subgraphs with high intragroup similarities were identified. An overview of the entire network is shown in Fig. 7A, and a subgraph with 11 highly similar nodes is shown in Fig. 7B. IC_50_ values for this subgraph ranged from 2.4 to 20.6 nM. This subgraph was from Cluster 31, and the average similarity of this cluster was 0.68. This further supports the finding that the CSNs analysis was consistent with the cluster analysis results. Based on the M4 backbone analysis, the presence of a fragment of 1,8-naphthyridin-4-one may enhance its potency and selectivity as a c-MET inhibitor.

### 3.6 Active cliff analysis

**Fig. 8:**
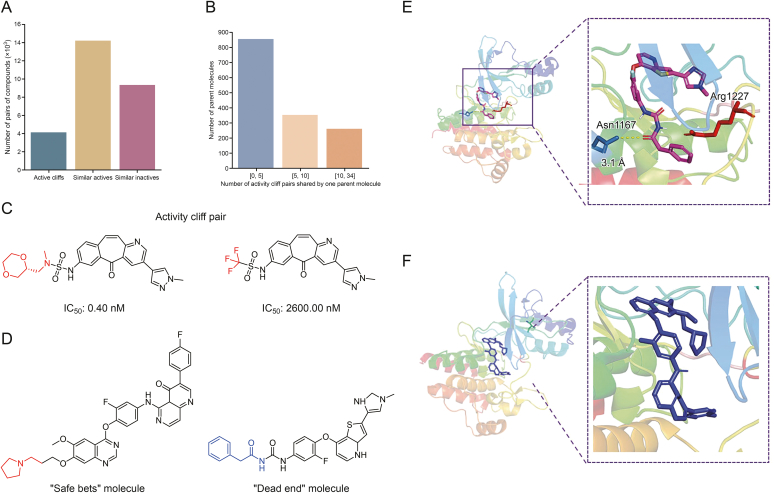
Classification of the typical active cliff pairs of compounds and their interactions with the c-mesenchymal-epithelial transition (c-MET) kinase domain. (A) Distribution of the different types of similar compounds in the dataset. (B) Compounds that are involved in activity cliffs and the corresponding distribution of activity cliff pairs per compound. (C) Example of an activity cliff pair: where the compound on the left has the half maximal inhibitory concentration (IC_50_) value of 0.40 nM, indicating high activity. The red functional group on the left is replaced by the red functional group on the right, the activity decreases significantly to the IC_50_ value of 2,600.00 nM. This demonstrates that the change in the functional group leads to a substantial change in the activity of the compound. (D) Examples of a “safe bet” compound and a “dead end” compound. Red and blue indicate positions where modifications resulted in gain and loss of activity, respectively. (E, F) Docking results of “dead end” (E) and “safe bets” (F) molecules.

After analyzing the core scaffold, it was crucial to further identify the structural modifications or substituents (R groups) associated with either diminished or enhanced activity of the c-MET inhibitors. To analyze this, we examined the pairs of compounds that displayed significant similarities (Tc > 0.6). These pairs were classified into three groups (Figs. 8A and B): 1) both compounds were active and shared a similar structure, 2) both compounds were inactive and shared a similar structure, and 3) cliff activity. Fig. 8C shows an example of an active cliff pair. Through this analysis, we identified a considerable number of cliff pair activities (*n* = 4,084).

Despite the vast chemical space available, a considerable number of compound pairs exhibit intriguing c-MET inhibitory activity. The exploration of the active cliffs revealed the top five scaffolds associated with the highest occurrence of such cliffs. These scaffolds were derived from the compounds in Clusters 3, 11, 7, 1, and 22. Although the appearance of active cliffs could not be completely avoided, certain scaffolds tended to produce active cliffs more frequently. The active cliff compounds were analyzed, and their distribution is shown in Fig. 8. Interestingly, 260 compounds were involved in cliff pair activity, with a range from 10 to 34 compounds in the opposing activity class. This classification refers to the combination of a reference inactive compound with N-paired actives or a reference active compound with N-paired inactive compounds. The former scenario was denoted as “safe bets”, whereas the latter was referred to as “dead ends”.

In total, 113 active compounds were identified in the “dead end” category. These compounds exhibited high similarity to inactive compounds 10–34, resulting in pairs of active cliffs. For instance, compounds from Cluster 3 were found in the active cliffs of 33 pairs (right side of Fig. 8D), where all activity loss modifications occurred in *N*-formyl-2-phenylacetamide at the positions marked in blue. This compound exhibits high activity (25 nM). However, when it underwent minor changes (conversion of O to S or imidazolidinethione), the activity loss reached multiple orders of magnitude (5,000 or 2,000 nM). It is advisable to employ complementary structure-based studies to gain insights into the role of these substituents in binding to c-MET, thus guiding further modifications.

The interaction patterns between the “safe bets” and “dead ends” with the c-MET kinase domains were performed through an example of molecular docking studies using the Protein Data Bank (PDB) ID 3RHK crystal structure. As illustrated in Fig. 8E, the representative docked ligand exhibits unfavorable donor-donor interactions between its terminal carboxylate groups and Arg1227’s guanidinium moiety. This finding aligns with previous structural studies [45] demonstrating that in the Asp-Phe-Gly (DFG)-out inactive state, Arg1227 forms stable hydrogen bonds with conserved acidic residues (e.g., Asp1222) while exposing its positively charged surface to the inhibitor-binding pocket. Notably, the negatively charged termini of the “dead ends” exhibit electrostatic repulsion with Arg1227’s charged surface, disrupting the optimal interaction network required for ATP-binding site inhibition. Comparatively, “safe bets” (in Fig. 8F) demonstrates good binding characteristics. Future studies would integrate multi-algorithm ensemble docking with more sampled conformations, free energy calculations and experimental validation.

### 3.7 Key structural alerts of c-MET inhibitors

**Fig. 9:**
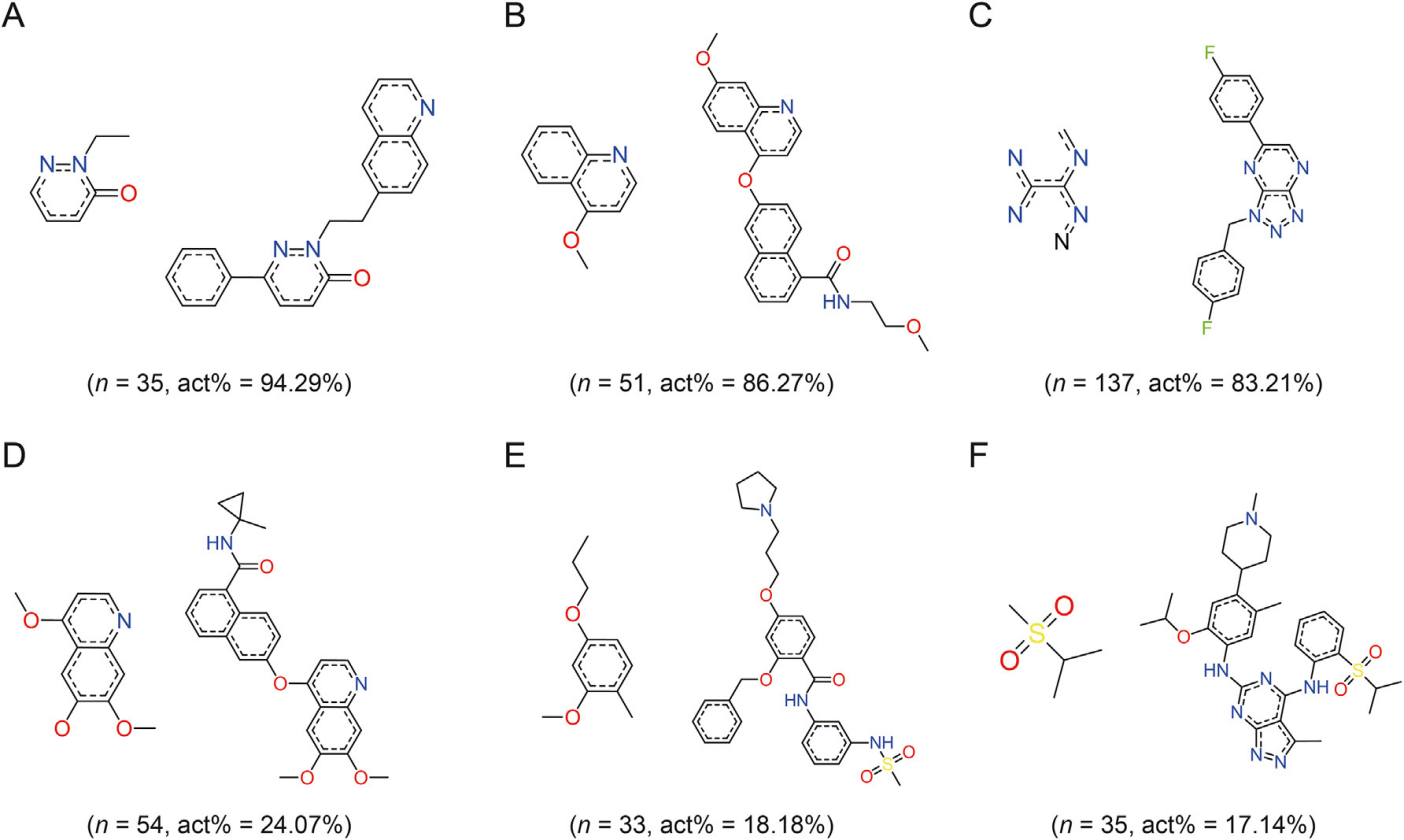
Key structural alerts of c-mesenchymal-epithelial transition (c-MET) inhibitor. The left side of each pair of compounds represents the substructure and the right side represents the compound containing this substructure. act%: proportion of active compounds.

Analyzing the chemical structural fragments related to the c-MET activity is crucial after analyzing the scaffolds and functional groups of c-MET inhibitors. We used the “Bioalerts” package in Python to analyze structural fragments related to activity [46]. The fragments containing ≥30 compounds were selected for analysis, and six structures were listed (Fig. 9). The first three structural motifs serve as potential starting points for the design of c-MET-active compounds and can be incorporated during structural modifications (Figs. 9A–C). Conversely, the presence of the last three structural fragments was avoided when designing the c-MET inhibitors (Figs. 9D–F).

According to the results analyzed by “Bioalerts”, pyridazinones were identified as structures favorable for c-MET inhibition. Among the 35 compounds analyzed, the active structures accounted for 94.29% (Fig. 9A). Previous studies have shown that the structures containing this fragment are strong inhibitors of c-MET, even at the nanomolar level [[47], [48], [49], [50]]. Quinolines constitute a large class of c-MET inhibitors [[51], [52], [53]]. However, their inhibitory activities were closely related to the functional group fragments to which they were attached (Figs. 9B and D). The activity was 86.27% for the 51 compounds with a core structure of 4-*en*-benzyloxyquinoline. When the core structure was 4,6,7-trimethoxyquinoline, the activity decreased to 24.07%, which was not favorable for c-MET inhibition. Fig. 9C shows a structure resembling that of a triazole, representing the structural resolution of triazole pyrazine. Numerous reports have also highlighted c-MET inhibition by this core structure [54,55], with active molecules accounting for 83.21% of the 137 compounds analyzed. The core structure shown in Fig. 9E is 1-methoxy-2-methyl-4-proxybenzene, and a simple benzene ring-connecting scaffold has little effect on c-MET inhibition. In addition, the isopropyl (methyl) dimethylene-l6-sulfone moiety contributed negligibly to the overall activity, accounting for only 17.14% of the total activity.

### 3.8 Physicochemical and structural guidelines for optimizing activity

To elucidate the distinct distribution patterns of active and inactive compounds within the chemical space and translate them into rules or guidelines, a decision tree model was constructed using all available compounds (Fig. S6). Despite limiting the depth to broader rules, the decision tree achieved a high training accuracy of 87.92%, confirming the successful segregation of active and inactive compounds. Anchor-exp was applied to the complete dataset, yielding 652 anchors (rules) with the top ten rules in terms of coverage, as highlighted in Table 1.

**Table 1:** General physicochemical and structural anchors (rules) of small molecule to predict c-mesenchymal-epithelial transition (c-MET) inhibition activity.

Rule	Anchor	Precision	Coverage	Number of compounds	Number of actives	Percentage of actives (%)	Number of inactives
1	NumHAcceptors > 6.00 and fr_pyridine > 1.00	0.86	0.11	241	195	80.91	46
2	NOCount > 8.00 and BCUT2D_MRHI ≤ 7.17 and PEOE_VSA9 > 28.24	0.86	0.11	251	208	82.87	43
3	BCUT2D_MWLOW ≤ 10.14 and fr_Ar_N > 5.00	0.91	0.09	210	179	85.24	31
4	fr_pyridine > 1.00 and fr_bicyclic > 1.00	0.82	0.09	197	164	83.25	33
5	BCUT2D_MWLOW ≤ 10.03 and PEOE_VSA5 > 0.00	0.85	0.08	156	126	80.77	30
6	BCUT2D_MWLOW ≤ 10.08 and fr_Ar_N > 5.00	0.93	0.05	101	89	88.12	12
7	fr_pyridine > 1.00 and NumAromaticHeterocycles > 3.00	0.90	0.05	101	86	85.15	15
8	fr_pyridine > 1.00 and PEOE_VSA5 > 0.00	0.83	0.04	96	83	86.46	13
9	fr_pyridine > 1.00 and SMR_VSA3 > 24.80	0.91	0.03	73	65	89.04	8
10	fr_pyridine > 1.00 and fr_Ar_N > 5.00	0.94	0.03	55	52	94.55	3

NumHAcceptors: number of hydrogen bond acceptors; fr_pyridine: number of pyridine rings; NOCount: number of nitrogens and oxygens; BCUT2D: two-dimensional (2D) Burden matrix with molar refractivity (MR) high eigenvalue; PEOE_VSA9: Molecular Operating Environment (MOE) Charge Valence Surface Area (VSA) Descriptor 9; BCUT2D_MWLOW: 2D Burden matrix with molecular weight low eigenvalue; fr_Ar_N: number of aromatic nitrogens; fr_bicyclic: number of bicyclic; PEOE_VSA5: MOE charge VSA descriptor 5; NumAromaticHeterocycles: number of aromatic heterocycles; SMR_VSA3: MOE MR VSA descriptor 3.

Among the top ten rules for the number of pyridine rings (fr_pyridine), the number of aromatic nitrogen atoms (fr_Ar_N) emerged as a descriptor. Interestingly, the presence of pyridine rings (fr_pyridine >1.00) was exclusively observed in the rules pertaining to the active compounds. The largest rule for active molecules encompassed a relatively static number of compounds (*n* = 241, 11% of the full dataset) and demonstrated remarkably high precision (80.91%). This rule states that an active compound comprises at least one pyridine ring and has more than 6.00 NumHAcceptors. The functionalities exposed by PEOE_VSA9, PEOE_VSA5, and SMR_VSA3, which are Molecular Operating Environment (MOE)-like approximate molecular surface area descriptors, also constitute important conditions for the active molecules. Notably, the decision-tree model precisely indicated the key structural features required to constitute active c-MET inhibitor molecules, including at least three aromatic heterocycles, five aromatic nitrogen atoms, and eight nitrogen−oxygen atoms. According to literature reports [[47], [48], [49]], the nitrogen atoms in quinoline and azaindoles can act as key binding elements in the kinase hinge region. By modifying the aromatic groups, it was possible to adjust the π−stacking interactions with Tyr1230, thereby enhancing the activity of the drug. These discoveries provide important guidance for the design of effective c-MET inhibitors.

## 4 Conclusion

In this study, we provide a systematic and comprehensive exploration of the chemical space of small-molecule c-Met inhibitors and offer crucial insights into their SARs and structural rule landscape. We collected and curated the largest and highest-quality publicly available datasets, which included 2,278 unique molecules. Drug-like property analysis and chemical space visualization demonstrated that although active compounds have more diverse chemical structures or scaffolds than inactive compounds, general physicochemical properties may not be the primary determinants of c-Met inhibitory activity. We employed chemoinformatics methods, including hierarchical clustering and CSNs, to discover commonly used backbones of c-MET inhibitors, such as M5, M7, and M8. Furthermore, we extracted all activity cliffs present in the dataset, highlighting a series of “dead ends” and “safe bets” related to the gain or loss of activity, and revealed their potential mechanisms of action through molecular docking. In particular, this study highlights that the pyridazinone structure is essential for the c-MET inhibitory activity. While the activity of quinolines is closely related to the functional groups to which they are attached, further SAR analyses revealed potential directions for optimizing c-MET inhibitors, suggesting that attention should be paid to the selection of core structures and fine-tuning of functional groups in drug design to improve inhibitory potency and selectivity. Notably, the decision-tree model precisely indicated the key structural features required to constitute active c-MET inhibitor molecules, including at least three aromatic heterocycles, five aromatic nitrogen atoms, and eight nitrogen−oxygen atoms. This discovery provides important guidance for the design of effective c-MET inhibitors. Finally, considering the rapid growth in the number of c-Met inhibitors, it is imperative to continuously curate and update the dataset.

## CRediT authorship contribution statement

**Jing Zhang:** Writing – original draft, Formal analysis, Data curation. **Mingming Zhang:** Software, Investigation, Data curation. **Weiran Huang:** Software, Investigation, Data curation. **Changjie Liang:** Data curation. **Wei Xu:** Data curation. **Jinghua Zhang:** Data curation. **Jun Tu:** Formal analysis. **Innocent Okohi Agida:** Writing – review & editing. **Jinke Cheng:** Writing – review & editing, Supervision. **Dong-Qing Wei:** Resources. **Buyong Ma:** Resources. **Yanjing Wang:** Writing – review & editing, Funding acquisition, Formal analysis. **Hongsheng Tan:** Writing – review & editing, Supervision, Funding acquisition, Conceptualization.

## Data availability

RDkit, Numpy, Scikit-learn, Pandas, Matplotlib, and Seaborn are freely available as Anaconda and/or pip packages (Python). The Anaconda/Jupyter notebook is available for download and installation at https://docs.anaconda.com/anaconda/install. All the raw data are available in the public domain (peer-reviewed publications, ChEMBL, and patents). Access to the full dataset and analysis scripts will be granted upon request.

## Declaration of competing interest

The authors declare that there are no conflicts of interest.
