# Supplementary material for: Scaffold and SAR studies on c-MET inhibitors using machine learning approaches
Source: J Pharm Anal. 2025 Apr 10;15(6):101303. doi: 10.1016/j.jpha.2025.101303 (PMC12268054; doi:10.1016/j.jpha.2025.101303)
Supplement: Multimedia component 1 [file mmc1.docx]

**Supplementary Materials**

**Scaffold and SAR Studies on c-MET Inhibitors Using Machine Learning Approaches**

**Contents:**

**Fig. S1** c-Mesenchymal-epithelial transition (c-MET) kinase and its binding mode with small-molecule inhibitors ---------------------------------------------------------------------------------------2

**Fig. S2** Historical perspective on trends in the overall chemistry of c-MET inhibitors----------3

**Fig. S3**. Absorption, Distribution, Metabolism, Excretion, and Toxicity (ADMET) attribute prediction of active and inactive compounds. --------------------------------------------------------- 4

**Fig. S4** Variation of silhouette scores with number of clusters--------------------------------------4

**Fig. S5** Intracluster similarity distribution of the 35 clusters obtained from the full dataset---------5

**Fig.S6** Decision Tree Model------------------------------------------------------------------------------6

**Table S1** List of published journals for all compounds ------------------------------------------------7

**Table S2** Scaffolds from clustering ----------------------------------------------------------------------8


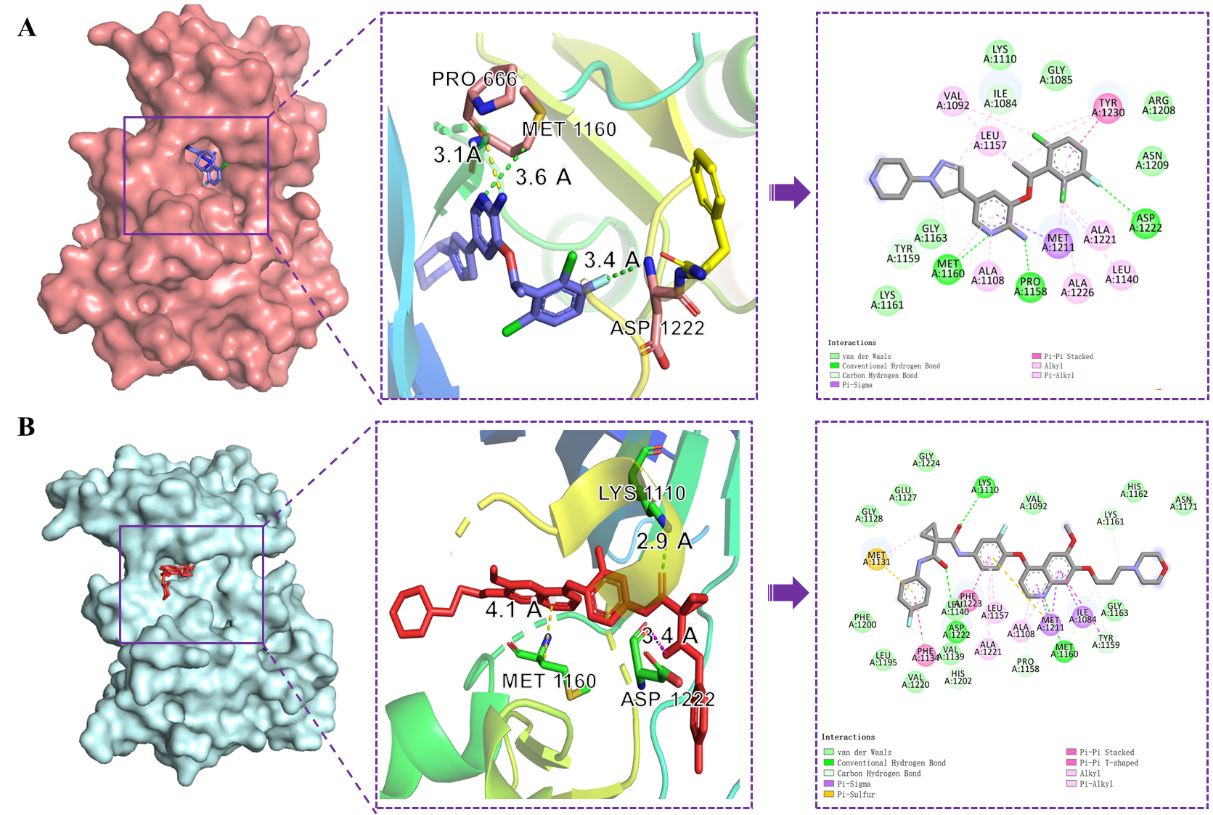


**Fig. S1**. c-Mesenchymal-epithelial transition (c-MET) kinase and its binding mode with small-molecule inhibitors. (A) Docking result of Crizotinib (PDB code: 2WGJ); (B) Binding result of Cabozantinib (PDB code: 3LQ8). PDB: Protein Data Bank.


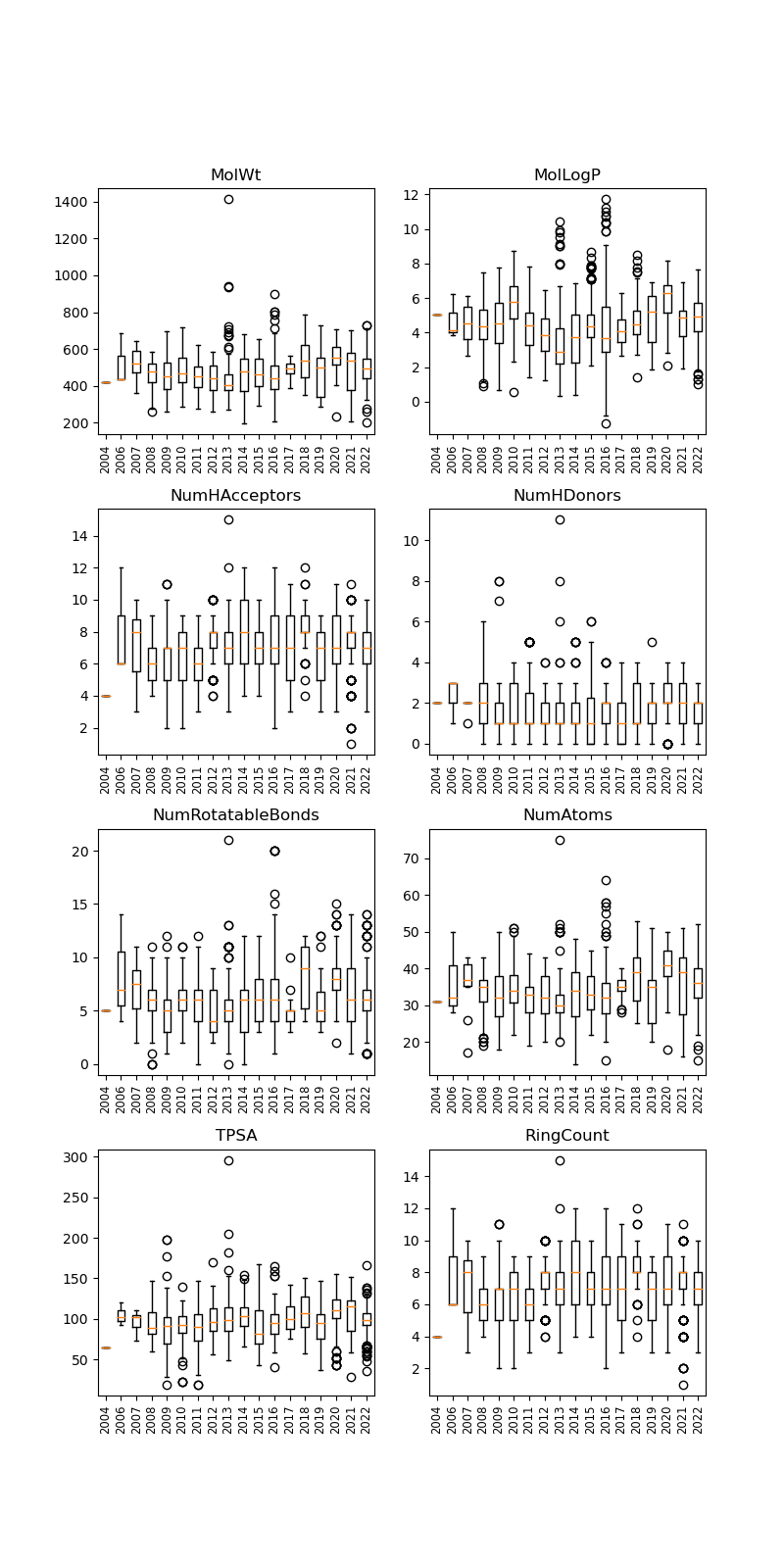


**Fig.S2**. Historical perspective on trends in the overall chemistry of c-Mesenchymal-epithelial transition (c-MET) inhibitors.

**
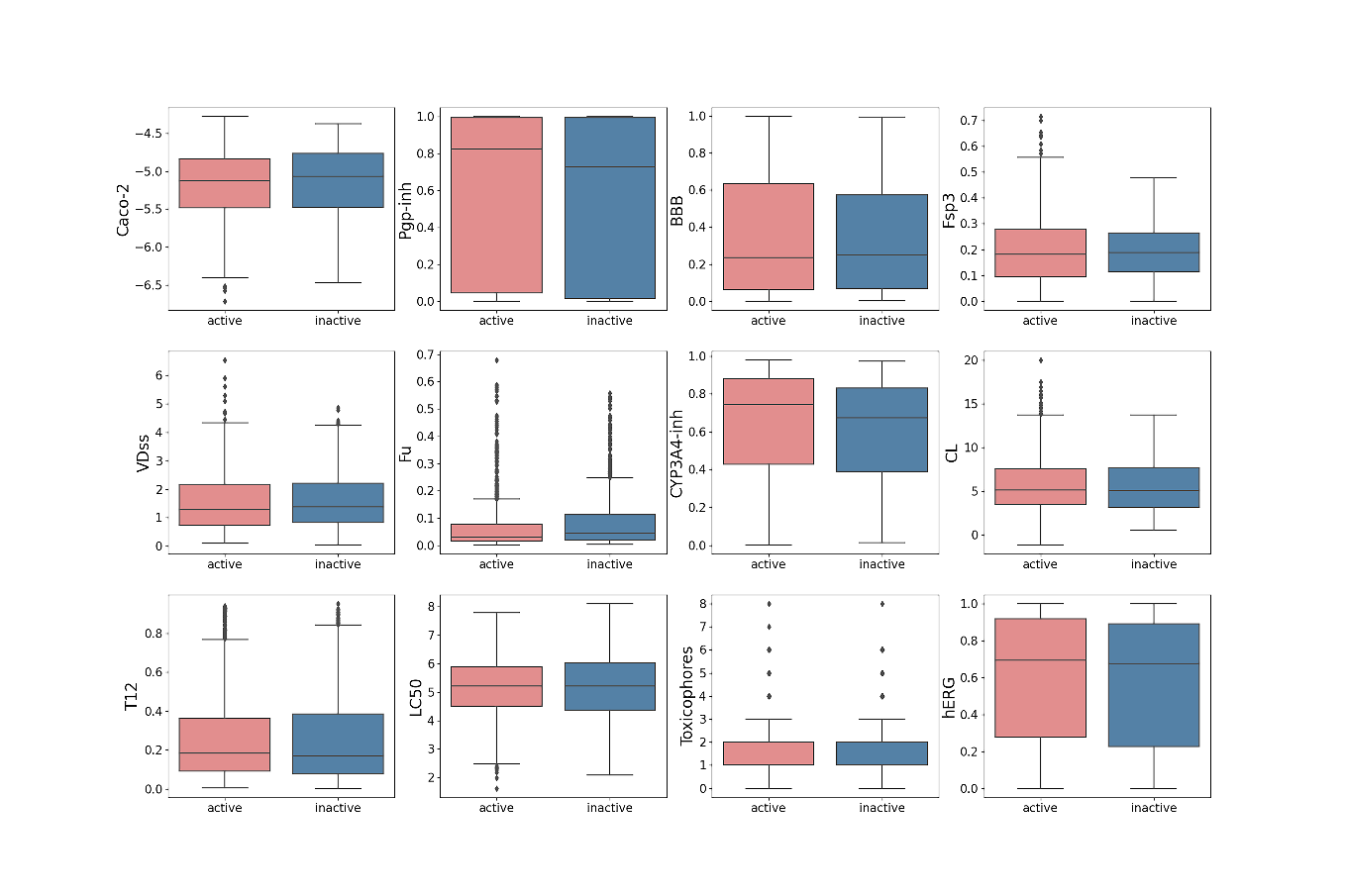
**

**Fig. S3**. Absorption, Distribution, Metabolism, Excretion, and Toxicity (ADMET) attribute prediction of active and inactive compounds. BBB: Blood brain barrier penetration; CL: clearance; CYP3A4: Cytochrome P450 protein 3A4; Fu: Fraction unbound in plasma; Fsp3: Fraction of sp3-hybridized carbons; hEGR: hERG (the human Ether-à-go-go-Related Gene) Blockers; LC_50_: Lethal concentration 50; Pgh-inh: probability of Pgp substrate inhibitor; QED: quantitative estimate of drug-likeness; T_1/2_: half-life; VD: volume of distribution.


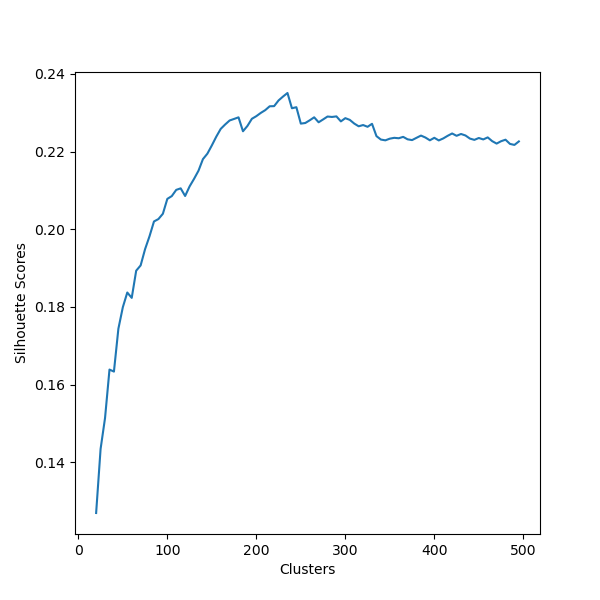


**Fig. S4**. Variation of silhouette scores with number of clusters.


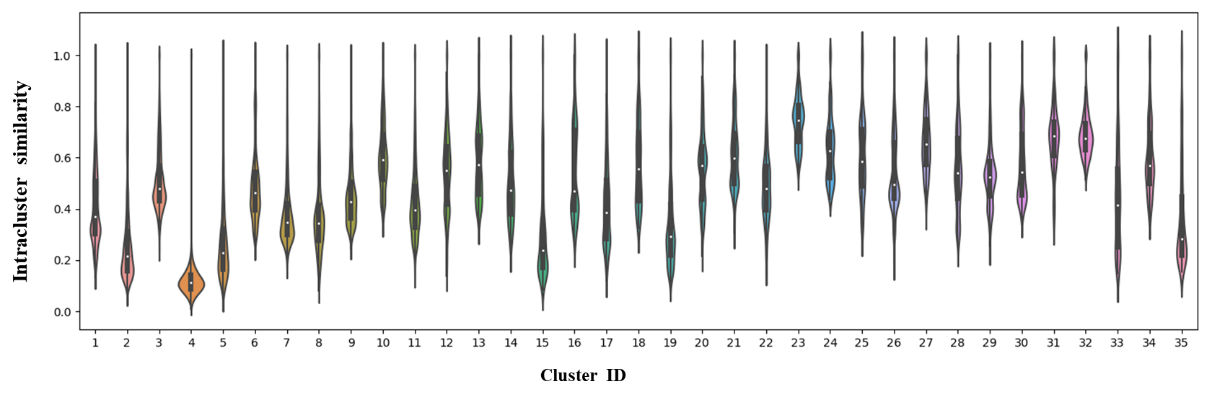


**Fig. S5**. Intracluster similarity distribution of the 35 clusters obtained from the full dataset. The cluster with the lowest number of 18 compounds (cluster 25) still had at least 23 compounds.


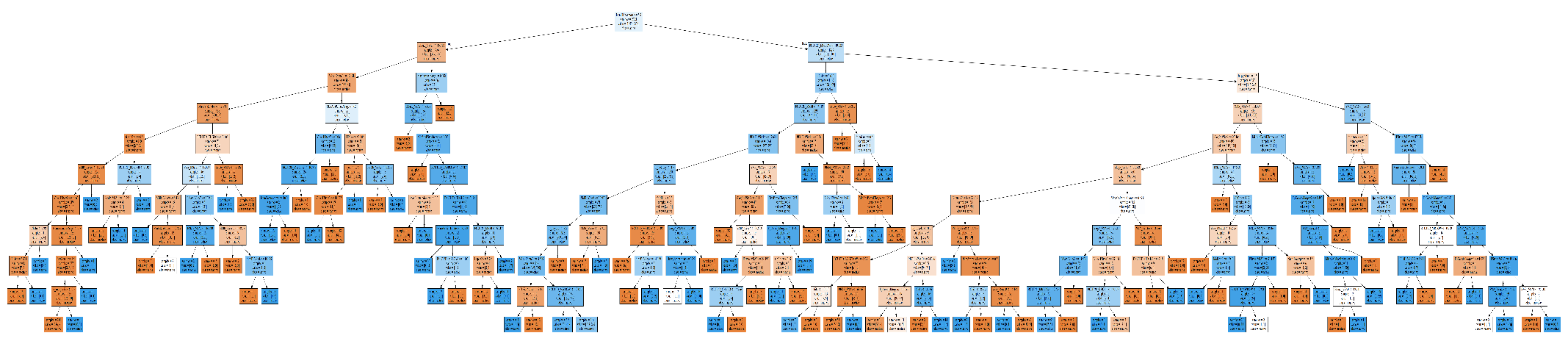


**Fig.S6**. Decision Tree Model.

**Table S1**. List of published journals for all compounds

| Journal Name Abbreviation | Impact Factor | JCR Ranking | Compound frequency | Percent (%) |
| --- | --- | --- | --- | --- |
| Bioorg. Med. Chem. Lett | 2.94 | Q4 | 656 | 28.80 |
| J Med Chem | 8.039 | Q1 | 592 | 25.99 |
| Eur J Med Chem | 7.088 | Q1 | 440 | 19.32 |
| Bioorg. Med. Chem | 3.461 | Q3 | 140 | 6.15 |
| ACS Med. Chem. Lett | 4.632 | Q2 | 79 | 3.47 |
| Anticancer Agents Med Chem | 2.527 | Q4 | 76 | 3.34 |
| Bioorg Chem | 5.307 | Q2 | 29 | 1.27 |
| Frontiers in Chemistry | 5.545 | Q3 | 21 | 0.92 |
| J. Nat. Prod | 4.803 | Q2 | 19 | 0.83 |
| Molecules | 4.927 | Q2 | 4 | 0.18 |
| Proc. Natl. Acad. Sci. U.S.A | 12.779 | Q1 | 3 | 0.13 |
| RSC Med Chem | 3.47 | Q3 | 1 | 0.04 |
| Biochem Pharmacol | 6.1 | Q2 | 1 | 0.04 |
| No | – | – | 176 | 7.73 |

JCR: Journal Citation Reports; No: no journal label information; –: no data.

**Table S2**. Scaffolds from clustering.

| **Cluster ID** | **MCS pattern SMARTS** | **comp. N** | **actives. %** | **ICS med.** | **ICS avg.** | **ICS min.** | **IC_50_**  **Avg (nM).** | **IC_50_**  **Med**  **(nM).** | **IC_50_**  **Min**  **(nM).** |
| --- | --- | --- | --- | --- | --- | --- | --- | --- | --- |
| 1 | [#6]:[#6]:[#7]:[#6]:[#6] | 127 | 68.5 | 0.37 | 0.41 | 0.13 | 1036.88 | 26 | 0.2 |
| 2 | [#6]1:[#6]:[#6]:[#6]:[#6]:[#6]:1 | 109 | 71.6 | 0.22 | 0.27 | 0.07 | 509.28 | 25 | 0.43 |
| 3 | [#6]:[#6]:[#6]:[#6](-[#8]-[#6]1:[#6]:[#6]:[#6]:[#6]:[#6]:1):[#6]:[#6]:[#7] | 119 | 37.8 | 0.48 | 0.51 | 0.23 | 485.80 | 130 | 1 |
| 4 | [#6]:[#6] | 203 | 17.2 | 0.11 | 0.13 | 0.01 | 9384.94 | 3900 | 0.5 |
| 5 | [#6]1-[#6]-[#6]-[#6]-[#6]:[#6]-1 | 76 | 100.0 | 0.23 | 0.27 | 0.06 | 2.97 | 1.39 | 0.03 |
| 6 | [#6]1:[#6]:[#6]:[#6]:[#6]:[#6]:1-[#7]-[#6]1:[#7]:[#6](-[#7]-[#6]):[#7]:[#6]:[#6]:1-[#17] | 77 | 79.2 | 0.46 | 0.49 | 0.25 | 92.93 | 21 | 1.9 |
| 7 | [#6]:[#6]:[#6]-[#8]-[#6]1:[#6]:[#6]:[#6]:[#6]:[#6]:1 | 116 | 57.8 | 0.35 | 0.38 | 0.17 | 377.79 | 57.65 | 0.7 |
| 8 | [#6]1(-[#6@@H]):[#6]:[#6]:[#6]:[#6]:[#6]:1 | 112 | 49.1 | 0.34 | 0.37 | 0.08 | 1096.05 | 120 | 0.32 |
| 9 | [#6]-[#8]-[#6]1:[#6]:[#6](:[#6]):[#6](:[#7]:[#6]):[#6]:[#6]:1-[#8] | 99 | 45.5 | 0.43 | 0.46 | 0.25 | 583.38 | 130 | 1.32 |
| 10 | [#6](:[#7]:[#6]):[#6]:[#6]-[#8]-[#6]1:[#6]:[#6]:[#6](-[#7]-[#6]2:[#7]:[#6]:[#6]:[#6]3:[#7H]:[#6]:[#6](-[#6]4:[#6]:[#6]:[#6]:[#6]:[#6]:4):[#6](=[#8]):[#6]:2:3):[#6]:[#6]:1 | 68 | 91.2 | 0.59 | 0.61 | 0.34 | 36.08 | 15.55 | 3.5 |
| 11 | [#6]1:[#6]:[#6]:[#6](:[#7]):[#6](:[#6]):[#6]:1 | 113 | 78.8 | 0.40 | 0.42 | 0.14 | 156.65 | 16 | 0.13 |
| 12 | [#6]1:[#6]:[#6](-[#6](:[#6]):[#6]):[#6]:[#7]:[#6]:1 | 66 | 59.1 | 0.55 | 0.54 | 0.14 | 496.94 | 66 | 0.2 |
| 13 | [#6]-[#6]1:[#6]:[#6]:[#6]2:[#7]:[#6]:[#6]:[#6]:[#6]:2:[#6]:1 | 37 | 89.2 | 0.57 | 0.59 | 0.33 | 578.31 | 10.6 | 2.8 |
| 14 | [#6]:[#6](-[#6](=[#8])-[#7]-[#6]1:[#6]:[#6]:[#6](-[#8]-[#6]:[#6]:[#6]:[#7]:[#6]):[#6]:[#6]:1):[#6] | 38 | 36.8 | 0.47 | 0.51 | 0.23 | 323.14 | 119.2 | 15 |
| 15 | [#6]1:[#6]:[#6]:[#6]:[#6]:[#6]:1 | 54 | 20.4 | 0.24 | 0.30 | 0.08 | 2999.94 | 860 | 20 |
| 16 | [#6]-[#8]-[#6]1:[#6]:[#6](:[#7]:[#6]):[#6](:[#6]-[#8]-[#6]2:[#6]:[#6]:[#6]:[#6]:[#6]:2):[#6]:[#6]:1 | 39 | 0.0 | 0.47 | 0.54 | 0.26 | 8342.31 | 5000 | 160 |
| 17 | [#6]1:[#6]:[#6]:[#6]:[#6](:[#7]:[#6]:[#6]):[#6]:1 | 65 | 29.2 | 0.39 | 0.42 | 0.12 | 4315.55 | 650 | 0.72 |
| 18 | [#6]-[#7]-[#6]1:[#6]:[#6]:[#6](-[#8]-[#6]2:[#6]:[#6]:[#7]:[#6]3:[#6]:2-[#8]-[#6]2:[#6]:[#6]:[#6]:[#6]:[#6]:2-[#7]-3):[#6]:[#6]:1 | 25 | 84.0 | 0.56 | 0.58 | 0.32 | 47.78 | 31.9 | 3.7 |
| 19 | [#6]:[#7]:[#6]1:[#6]:[#6]:[#6]:[#6]:[#6]:1 | 63 | 90.5 | 0.29 | 0.34 | 0.11 | 37.23 | 7 | 0.6 |
| 20 | [#6]-[#6](=[#8])-[#7]-[#6]1:[#6]:[#6]:[#6](-[#8]-[#6]:[#6]:[#6]):[#6]:[#6]:1 | 60 | 31.7 | 0.57 | 0.56 | 0.22 | 1454.28 | 390 | 0.61 |
| 21 | [#6]1:[#7]:[#6]:[#6]:[#6](-[#8]-[#6]:[#6]:[#6]:[#6]-[#7]-[#6](=[#8])-[#6]:[#6]=[#8]):[#6]:1:[#6]:[#6] | 50 | 78.0 | 0.60 | 0.61 | 0.30 | 101.05 | 51 | 1 |
| 22 | [#6]1:[#6]:[#7]:[#6]2:[#6]:[#6]:[#6]3:[#6]:[#6]:[#6]:[#6]:[#6]:3:[#6]:[#6]:2:[#6]:1 | 97 | 45.4 | 0.48 | 0.49 | 0.15 | 908.97 | 140 | 0.2 |
| 23 | [#8]-[#6]1:[#6]:[#6]2:[#7]:[#6]:[#6]:[#6](-[#8]-[#6]3:[#6]:[#6]:[#6](-[#7](-[#6](=[#8])-[#6]4(-[#6](-[#7])=[#8])-[#6]-[#6]-4)-[#6]4:[#6]:[#6]:[#6](-[#9]):[#6]:[#6]:4):[#6]:[#6]:3):[#6]:2:[#6]2:[#6]:1-[#8]-[#6]-[#6]-[#8]-2 | 32 | 100.0 | 0.75 | 0.74 | 0.53 | 50.00 | 50 | 50 |
| 24 | [#6]1:[#6]:[#6]:[#6]:[#6](-[#6]2:[#6]:[#6]:[#6](=[#8]):[#7](-[#6]-[#6@@H]3-[#6]-[#7](-[#6]4:[#7]:[#6]:[#6]:[#6]:[#7]:4)-[#6]-[#6]-[#8]-3):[#7]:2):[#6]:1 | 30 | 93.3 | 0.63 | 0.63 | 0.44 | 44.63 | 12.5 | 2 |
| 25 | [#7]-[#6]:[#7]:[#6]1:[#6]:[#6]:[#6](-[#16]-[#6]2:[#7]:[#7]:[#6]3:[#6]:[#6]:[#6]:[#7]:[#7]:2:3):[#6]:[#6]:1 | 23 | 17.4 | 0.59 | 0.61 | 0.31 | 717.70 | 216 | 2 |
| 26 | [#6]1:[#6]:[#6]:[#6]:[#6]:[#6]:1-[#7]-[#6]1:[#7]:[#6](-[#7]):[#7]:[#6]:[#6]:1 | 37 | 16.2 | 0.49 | 0.55 | 0.20 | 537.70 | 220 | 6.9 |
| 27 | [#6]-[#8]-[#6](=[#8])/[#6]=[#6]/[#6]1:[#6]:[#6]:[#6]:[#6]:[#6]:1 | 27 | 0.0 | 0.65 | 0.66 | 0.39 | 6662.96 | 6100 | 1000 |
| 28 | [#6]-[#8]-[#6](:[#6]):[#6]:[#6]1:[#6]:[#6]:[#6]:[#6]:[#7]:1 | 38 | 71.1 | 0.54 | 0.56 | 0.25 | 130.71 | 17 | 2.02 |
| 29 | [#7]-[#6]1:[#6]:[#6]:[#6](-[#8]-[#6]2:[#6]:[#6]:[#7]:[#6]3:[#7H]:[#6]:[#6]:[#6]:2:3):[#6]:[#6]:1 | 58 | 50.0 | 0.53 | 0.53 | 0.23 | 1216.78 | 98.5 | 1.3 |
| 30 | [#6]1:[#6]:[#6]:[#7]2:[#7]:[#6]:[#7]:[#6](-[#8]-[#6]3:[#6]:[#6]:[#6](-[#7]-[#6]=[#8]):[#6]:[#6]:3-[#9]):[#6]:1:2 | 62 | 37.1 | 0.54 | 0.58 | 0.35 | 271.23 | 157 | 0.71 |
| 31 | [#7]-[#6]1:[#7]:[#6]:[#7]:[#6]2:[#7H]:[#6]:[#6](-[#6]3:[#6]:[#6]:[#6](-[#7]-[#6](=[#8])-[#6]4:[#6]:[#7]:[#6]:[#6](-[#6]5:[#6]:[#6]:[#6]:[#6]:[#6]:5):[#6]:4=[#8]):[#6]:[#6]:3):[#6]:1:2 | 25 | 92.0 | 0.68 | 0.68 | 0.33 | 32.45 | 17 | 1.7 |
| 32 | [#8]-[#6]1:[#6]:[#6](-[#8]-[#6]-[#6]-[#6]-[#7]2-[#6]-[#6]-[#6]-[#6]-2):[#6]:[#6]:[#6]:1-[#6](=[#8])-[#7]-[#6]:[#6]:[#6]:[#6] | 40 | 15.0 | 0.68 | 0.69 | 0.51 | 2168.04 | 748.395 | 17.38 |
| 33 | [#6]1:[#6]:[#6]:[#6]:[#6](-[#6]-[#7]:[#6]=[#8]):[#6]:1 | 29 | 48.3 | 0.41 | 0.44 | 0.15 | 649.34 | 140 | 1 |
| 34 | [#6]-[#6](-[#6](:[#6]:[#6]:[#6]:[#7]:[#6]:[#6]):[#6])-[#7]1:[#7]:[#7]:[#6]2:[#7]:[#6]:[#6](-[#6](:[#6]):[#6]):[#7]:[#6]:1:2 | 27 | 100.0 | 0.57 | 0.60 | 0.36 | 4.78 | 0.7 | 0.3 |
| 35 | [#8]=[#6](-[#7]-[#6]1:[#6]:[#6]:[#6]:[#6]:[#6]:1)-[#6]-[#6] | 37 | 35.1 | 0.28 | 0.37 | 0.15 | 1571.72 | 289 | 10 |

MCS = Maximum common substructure (i.e. common 35 scaffold); ICS min = minimum intracluster similarity; ICS avg= average intracluster similarity; ICS med= median intracluster similarity.
